# Editorial: The need for pragmatic trials in genitourinary oncology

**DOI:** 10.3389/fonc.2025.1702188

**Published:** 2025-10-02

**Authors:** Mieke Van Hemelrijck, Piet Ost, Giorgio Gandaglia

**Affiliations:** ^1^ Faculty of Life Sciences and Medicine, Transforming Cancer Outcomes Through Research (TOUR), King’s College London, London, United Kingdom; ^2^ Department of Human Structure & Repair, Faculty of Medicine and Health Sciences, Ghent University, Ghent, Belgium; ^3^ Unit of Urology/Division of Oncology, Gianfranco Soldera Prostate Cancer Laboratory, IRCCS San Raffaele Scientific Institute, Vita-Salute San Raffaele University, Milan, Italy

**Keywords:** prostate cancer, bladder cancer, kidney cancer, pragmatic trial, real world evidence

The landscape of genitourinary (GU) oncology is rapidly evolving, driven by advances in molecular diagnostics, availability of novel systemic therapies such as immunotherapy, and the implementation of big-data and real-world data integration. Yet, traditional randomised controlled trials (RCTs), while foundational, often fall short in addressing the complexities of routine clinical practice (1, 2). This Research Topic brings together five diverse contributions that underscore the urgent need for pragmatic trials - those designed to reflect real-world settings, patient diversity, and clinical decision-making - in GU oncology.

## Bridging research and reality: trials within cohorts

In their cohort profile of the Graham Roberts Study, Russell et al. present the first Trials within Cohorts (TwiCs) design for bladder cancer (3). This innovative infrastructure enables longitudinal data collection and embedded randomised trials, with a focus on patient-reported outcomes (PROs) such as fatigue, depression, and quality of life. The study exemplifies how pragmatic designs can facilitate efficient recruitment, reduce burden on participants, and support future interventions—particularly in supportive care domains like mental wellbeing. The authors highlight the potential of TwiCs to overcome recruitment barriers and improve trial generalizability, especially in underrepresented populations (4).

## Registry-based randomized controlled trials: lessons from REAL-Pro

Anton et al., in their perspective on the REAL-Pro study, explore the utility of registry-based randomised controlled trials (RRCTs) in advanced prostate cancer. Using the electronic Prostate Cancer Australian and Asian Database (ePAD), the authors demonstrate how RRCTs can leverage existing infrastructure to conduct low-cost, high-impact trials. Despite the promise, REAL-Pro faced slow accrual, reflecting challenges in clinician equipoise and patient eligibility. The study underscores the importance of understanding stakeholder perspectives and designing trials that align with clinical workflows. Importantly, the authors advocate for qualitative research to identify barriers and optimise future pragmatic trial designs (5, 6).

## De-escalation and patient-centered care: the EORTC 2238 trial

Grisay et al. introduce the EORTC 2238 “De-Escalate” trial, a multicenter pragmatic trial assessing intermittent versus continuous maximal androgen blockade (MAB) in metastatic hormone-naïve prostate cancer. Using the PRECIS-2 framework (7) and a two-stage consent model (3), the trial exemplifies how pragmatic designs can reduce toxicity, enhance quality of life, and optimise resource use. The authors argue that intermittent therapy may offer comparable oncologic outcomes with fewer side effects, particularly in patients with deep PSA responses. The trial’s design reflects a shift toward patient-centred endpoints and real-world applicability, challenging the conventional paradigm of continuous treatment. It underscores the importance of prioritising quality of life alongside cancer control in the management of metastatic prostate cancer.

## Expanding the evidence base: immunotherapy in rare histologies

In a compelling case report, Nagahisa et al. describe a complete response to ipilimumab and nivolumab in a patient with advanced papillary renal cell carcinoma (pRCC) and inferior vena cava (IVC) tumour thrombus. This rare presentation, typically associated with poor prognosis, responded dramatically to immune checkpoint inhibitors, enabling deferred cytoreductive nephrectomy. The case highlights the potential of neoadjuvant immunotherapy in non-clear cell RCC subtypes and calls for pragmatic trials to validate such approaches. Given the rarity of pRCC with IVC involvement, traditional RCTs are unlikely to address this clinical scenario - further reinforcing the value of flexible, real-world trial designs (8).

## Diagnostic precision and treatment de-escalation: renal NETs

Wang et al. present a case series and literature review on renal neuroendocrine tumours (NETs), emphasising the diagnostic challenges and risk of overtreatment. These rare tumours often mimic renal cell carcinoma on imaging, leading to unnecessary radical nephrectomies. The authors advocate for improved preoperative diagnostics, including molecular imaging and biopsy, and suggest that nephron-sparing approaches may be appropriate in select cases. Their findings also underscore the need for pragmatic trials to evaluate diagnostic algorithms and conservative treatment strategies, particularly in rare GU malignancies.

Collectively, these five contributions illuminate the transformative potential of pragmatic trials in GU oncology. Whether through TwiCs, RRCTs, or adaptive designs, pragmatic trials offer a pathway to more inclusive, efficient, and patient-centred research. They enable the evaluation of standard-of-care interventions, facilitate real-world implementation, and prioritise outcomes that matter to patients. As the field moves toward precision oncology and value-based care, pragmatic trials will be essential in bridging the gap between evidence and practice. This Research Topic invites clinicians, researchers, and policymakers to reimagine trial design - not as a rigid protocol, but as a dynamic tool for improving outcomes in the real world.
